# Retrospective Analysis of 411 Cases of Spontaneous Portosystemic Shunt Complicated With Oesophageal and Gastric Variceal Bleeding in Cirrhotic Patients

**DOI:** 10.1155/cjgh/4623854

**Published:** 2026-02-03

**Authors:** Zhaoyi Chen, Jing Li, Xi Wang, Xuecan Mei, Yaxian Kuai, Huixian Li, Derun Kong

**Affiliations:** ^1^ Key Laboratory of Digestive Diseases of Anhui Province, Department of Gastroenterology, The First Affiliated Hospital of Anhui Medical University, Hefei, China, ahmu.edu.cn; ^2^ Xuancheng People’s Hospital, Xuancheng, Anhui, China

**Keywords:** EGVB, rebleeding diameter, SPSS

## Abstract

This study is aimed at evaluating the incidence and clinical characteristics of spontaneous portosystemic shunts (SPSSs) in cirrhotic patients with oesophageal and gastric variceal bleeding (EGVB) and investigating the impact of SPSSs on the course of EGVB. A retrospective analysis of 1110 cirrhotic patients with EGVB was conducted to determine SPSS incidence and characteristics and evaluate the impact of SPSS size and endoscopic treatments. The SPSS incidence was 94.26% (411/436), with gastric renal shunts (78.89%) being the most common. SPSSs were categorized by diameter as follows: S‐SPSS (≤ 5 mm), M‐SPSS (5–8 mm), and L‐SPSS (≥ 8 mm). A larger SPSS diameter was correlated with higher model for end‐stage liver disease (MELD) scores, international normalized ratios (INRs), total bilirubin (TB) levels, and portal vein thrombosis. During the 12‐month follow‐up, the L‐SPSS group had a lower rebleeding rate (3.38%) than the S‐SPSS (14.64%) and M‐SPSS (16.88%) groups. A larger SPSS diameter was associated with a 77% reduction in rebleeding risk (HR = 0.23, *p* = 0.016). In summary, a larger SPSS diameter is linked to worse liver function but reduced rebleeding risk in cirrhotic patients with EGVB.

## 1. Introduction

As a prevalent clinical disease, liver cirrhosis is characterized by chronic, debilitating, and irreversible features that significantly impact the quality of life of patients. The obstruction of portal vein blood flow and blood stasis in cirrhosis lead to increased portal vein pressure, causing intrahepatic and extrahepatic portosystemic shunts. As a compensatory mechanism for portal hypertension, a spontaneous portosystemic shunt (SPSS) forms, aiming to reduce portal vein pressure [1]. When this balance is disrupted, various complications of portal hypertension occur, particularly digestive tract bleeding, which has a rapid onset, a high frequency, and a high mortality rate, severely affecting the quality of life and survival rate of patients [2]. Studies have shown that an SPSS is present in 40%–70% of cirrhosis patients [3]. Nevertheless, research on the incidence of SPSS in patients with cirrhosis‐related oesophagogastric variceal bleeding (EGVB) and the relationship between different sizes of SPSSs and rebleeding is limited. The present study is aimed at evaluating the prevalence and clinical characteristics of SPSS in patients with EGVB and assessing the impact of SPSS on the disease course.

## 2. Materials and Methods

This single‐centre retrospective observational study involved 1110 patients with cirrhotic EGVB who were admitted to the First Affiliated Hospital of Anhui Medical University between January 2018 and March 2024. Among these patients, 436 patients underwent both endoscopy and enhanced computed tomography angiography (CTA) of the abdomen. The patient demographics, aetiology of liver disease, disease progression, complications, rebleeding, and treatments were documented and monitored.

### 2.1. Study Cohort and Data Collection

A total of 1110 cirrhosis patients admitted for gastrointestinal bleeding from January 2018 to March 2024 at the First Affiliated Hospital of Anhui Medical University were screened. The inclusion criteria were a diagnosis of cirrhosis and completion of endoscopy and abdominal enhanced CTA examination. The exclusion criteria included incomplete CT imaging for portal shunt assessment, a prior transjugular intrahepatic portal‐systemic shunt (TIPS) or surgical shunting, an expected survival of less than 6 months, conditions hindering hepatic encephalopathy (HE) assessment, and missing critical history information. Patients meeting the inclusion criteria and not meeting the exclusion criteria underwent a medical history review. The date of the CT imaging was considered the baseline. Detailed demographic characteristics, aetiology, course of liver disease, complications, rebleeding, and treatment measures were recorded for all included patients. Laboratory and clinical parameters were collected at baseline, and oesophagogastroduodenoscopy data, including time, treatment methods, and varix morphology, were also collected. Liver function was evaluated using the model for end‐stage liver disease (MELD) and Child‒Pugh scores at baseline. Follow‐up included all decompensation events, such as ascites, gastrointestinal bleeding due to portal hypertension, hepatorenal syndrome (HRS), spontaneous bacterial peritonitis, other infections, liver transplantation, death, or hepatocellular carcinoma (HCC) development from baseline.

### 2.2. Imaging and Endoscopy Data and Definitions

1. SPSS: abdominal enhanced CT imaging results were evaluated by two radiologists with expertise in abdominal diagnostic imaging, who simultaneously observed the patient’s images to assess the type and size of the SPSS. The SPSS is considered to originate from additional vessels, such as the superior mesenteric veins, inferior mesenteric veins, splenic veins, portal veins, renal veins, and inferior vena cava, and it is verified through sagittal and coronal reconstruction [1]. Patients were divided into the following three groups according to the maximum diameter of the SPSS: the small shunt group (S‐SPSS, ≤ 5 mm), the medium shunt group (M‐SPSS, 5–8 mm), and the large shunt group (L‐SPSS, ≥ 8 mm). In addition to the detailed imaging information of the SPSS, other imaging information, such as portal vein thrombosis (PVT), visceral vein thrombosis, spleen size, portal vein diameter, splenic vein diameter, and ascites, was collected.
2. Gastrorenal shunt (GRS): GRS refers to the abnormal passage of blood from the short gastric vein or left gastric vein draining directly into the left renal vein, which is commonly exhibited on imaging as an anomalous vascular pathway extending from the gastric to renal regions [2].
3. Splenorenal shunt (SRS): SRS refers to the abnormal drainage of blood from the splenic vein to the left renal vein. Typically, this shunt is achieved through an abnormal venous channel formed between the splenic vein and left renal vein, and it is visible on imaging as an anomalous vascular channel extending from the spleen to the kidney [3].
4. Mixed vein shunt (MVS): MVS refers to the presence of multiple types of spontaneous portal‐systemic shunts simultaneously. On imaging, MVS manifests as the coexistence of multiple abnormal vascular channels [4].
5. Umbilical vein shunt (UVS): UVS refers to an abnormal blood flow pathway formed through the umbilical vein without surgical intervention [5].
6. Gastroscopy: the oesophagogastric varix type, varix number, varix diameters, presence of red colour signs, and treatment methods, including the number of band ligation loops, amount of sclerosant used, tissue adhesive dose, and complications, were recorded. In the present study, endoscopic procedures were categorized into two independent variables: endoscopic surveillance frequency and endoscopic intervention frequency. The endoscopic surveillance frequency was defined as the number of diagnostic endoscopic examinations performed solely to assess variceal characteristics—such as the size, number, and presence of red colour signs—without any therapeutic intervention. In contrast, the endoscopic intervention frequency refers to the number of endoscopic sessions involving therapeutic procedures, including endoscopic injection sclerotherapy (EIS), endoscopic variceal ligation (EVL), and cyanoacrylate injection. To accurately evaluate the prognostic impact of endoscopic therapy on the risk of rebleeding, these two variables were separately incorporated into the multivariate regression models. Additionally, to ensure cohort homogeneity and minimize potential selection bias, only patients admitted for their first documented episode of EGVB were included. A detailed record of any prophylactic endoscopic interventions prior to the index hospitalization (e.g., pre‐emptive EVL or EIS) was maintained and appropriately adjusted for in the statistical analysis.
7. Follow‐up: follow‐up was conducted in either inpatient or outpatient settings. The follow‐up data primarily included symptoms of gastrointestinal bleeding, such as haematemesis and melena, biochemical indicators, and CT imaging. During repeat gastroscopy, observation focused on whether varices were eliminated or alleviated, if they had disappeared or largely disappeared, the occurrence of new varices, the recurrence or varices, and treatment. The following information was also documented: whether patients experienced rebleeding, the time from initial treatment to rebleeding, the cause of bleeding, the occurrence of ectopic embolization, the location, the timing, and any concurrent complications.

### 2.3. Observation Indicators

The primary observation indicators included the incidence rate and clinical characteristics of EGVB in patients with cirrhosis and the effects of different SPSS sizes and endoscopic treatments. The secondary observation indicators were obtained from correlation analysis between the diameter of the SPSS and various clinical factors; comparison of liver and kidney function changes between patients at baseline and after 12 months; and assessment of risk factors for rebleeding.

### 2.4. Statistical Analysis

All the statistical analyses were performed using SPSS software (version 27, IBM, Armonk, NY, USA). Line graphs were plotted using Excel, and correlation heatmaps were created using R software. Nonparametric tests were used to analyse continuous variables, and the results are reported as the means ± standard deviations, medians, and ranges. Categorical variables are reported as numbers (%), and data were analysed using various statistical methods, including one‐way ANOVA, paired *t* tests, chi‐square tests, the Whitney *U* test, and the rank‐sum test, with frequency and percentage descriptions. Spearman correlation analysis was used to calculate correlation coefficients, denoted as “*r*.” A significance level of *p* < 0.05 was considered statistically significant.

## 3. Results

### 3.1. Study Population

**FIGURE 1:**
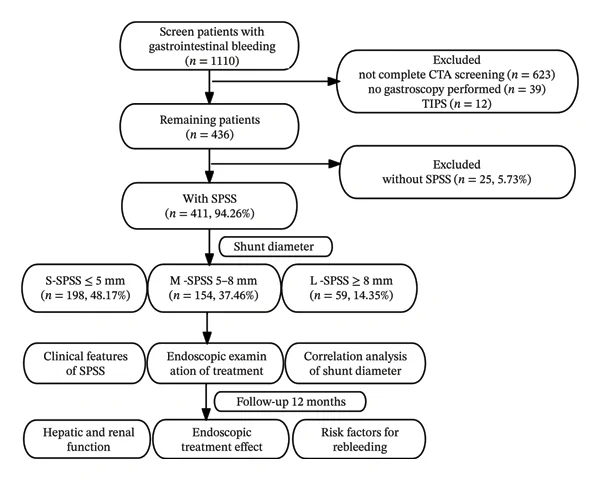
Flow diagram of participants in the study. TIPS: transjugular intrahepatic portosystemic shunt; L‐SPSS: large spontaneous portosystemic shunt; S‐SPSS: small spontaneous portosystemic shunt; M‐SPSS: medium spontaneous portosystemic shunt.

A total of 1110 patients with liver cirrhosis and EGVB were initially screened for the present study. After excluding patients who were elderly or had incomplete endoscopic examinations due to poor cardiopulmonary function (39 patients), patients who did not complete portal vein reconstruction (623 patients), and patients who had undergone TIPS (12 patients), the final cohort included 436 patients with liver cirrhosis and EGVB who underwent both endoscopic and abdominal enhanced portal venous vascular imaging scans (Figure 1).

### 3.2. Incidence and Types of SPSSs

Among the 436 patients included in the study, the median age was 53.57 years (range: 29–83 years), with 316 males (72.47%) and 120 females (27.52%). Among the included patients, 411 patients had SPSSs, with an incidence rate of 94.26% (411/436), including GRSs in 344 patients (78.89%), no SPSSs in 25 cases (5.73%), mixed SPSSs in 45 patients (10.32%), UVSs in 17 patients (3.89%), and SRSs in 5 cases (1.14%). In addition to EGVB, the distribution of other complications among different types of portosystemic shunts was as follows: in the GRS group, HE occurred in 4 patients (1.16%), bacteraemia occurred in 7 patients (2.03%), PVT occurred in 119 patients (34.59%), HCC occurred in 31 patients (9.01%), and HRS occurred in 1 patient (0.29%); in the MVS group, HE occurred in 1 patient (2.22%), PVT occurred in 29 patients (64.44%), and HCC occurred in 1 patient (2.22%); in the PVS group, PVT occurred in 1 patient (5.88%); and in the SRS group, HE and HCC were each observed in 1 patient (20.0%). A total of 1088 endoscopic examinations or re‐examinations were conducted, with 639 patients undergoing endoscopic treatment. During the 12‐month follow‐up, 60 patients (13.76%) experienced at least one rebleeding episode. Among them, 36 patients had HCC, and nine patients died during the follow‐up period.

### 3.3. Baseline Liver Function and SPSS Diameter

Analysis of the baseline characteristics, biochemical results, and imaging findings of the patients (Table 1) revealed significant differences in MELD scores among the three groups (8.90 ± 2.12 vs. 9.32 ± 2.47 vs. 10.86 ± 3.43; *p* < 0.001), suggesting a correlation between SPSS diameter and liver function. The international normalized ratio (INR) (*p* < 0.001) and total bilirubin (TB) (*p* = 0.016) also significantly differed among the three groups, indicating increases in the INR and TB with increasing SPSS diameter. Imaging data revealed that both the splenic vein diameter and velocity (*p* < 0.001) were smaller in the M‐SPSS group compared with the other groups. Additionally, the L‐SPSS group had a greater incidence of PVT (49.15%), although there was no significant difference among the three groups (*p* = 0.072). However, a significant difference in the incidence of PVT was observed between the L‐SPSS and M‐SPSS groups (32.46%) (*p* = 0.024), suggesting an increased likelihood of PVT with an SPSS diameter > 8 mm.

**TABLE 1:** Backgrounds of the patients.

Factors	With SPSS	Shunt diameter			*p*‐value
		≤ 5 mm	5–8 mm	> 8 mm	
	*n* = 411	*n* = 198	*n* = 154	*n* = 59	
Baseline general					
Median age, years (range)	53.59 ± 11.13	55.10 ± 11.48	52.46 ± 11.09	51.47 ± 9.31	0.052
Sex (male/female)	297/114 (72.26/27.73%)	137/61 (69.19/30.8%)	113/41 (73.37/26.62%)	48/11 (81.35/18.64%)	0.066
History of liver cirrhosis (years)	5.85 ± 5.53	5.52 ± 5.71	5.84 ± 4.82	7.02 ± 6.50	0.096
Etiology (HBV/unknown/PBC/alcohol/others)	(247/49/46/45/24)	(111/28/28/24/7)	(102/18/12/15/7)	(38/6/6/6/3)	—
Liver disease‐related deaths, *n* (%)	8 (0.19%)	4 (2.00%)	2 (1.29%)	2 (3.38%)	0.613
HCC	33 (8.02%)	14 (7.07%)	12 (7.79%)	7 (11.86%)	0.512
Baseline scores					
MELD score	9.39 ± 2.59	8.90 ± 2.12	9.32 ± 2.47	10.86 ± 3.43	< 0.001^∗∗^
Child–Pugh score	6.77 ± 0.94	6.72 ± 0.85	6.80 ± 1.01	6.83 ± 1.03	0.67
Child–Pugh (class A/B/C)	119/286/6	80/116/2	68/82/4	19/39/1	—
Baseline laboratory					
WBC (× 10^12^/L)	3.24 ± 1.88	3.05 ± 1.60	3.36 ± 2.03	3.58 ± 2.28	0.14
Hemoglobin (g/L)	98.81 ± 65.07	100.06 ± 90.29	98.31 ± 26.68	96.05 ± 28.28	0.082
Platelet (× 10^9^/L)	89.92 ± 84.07	86.65 ± 74.79	93.18 ± 94.60	91.96 ± 84.38	0.784
INR	1.17 ± 0.17	1.14 ± 0.13	1.17 ± 0.18	1.25 ± 0.22	< 0.001^∗∗^
Baseline laboratory					
ALb (g/L)	34.72 ± 4.64	34.83 ± 4.68	34.71 ± 4.78	34.40 ± 64.85	0.844
TB (μmol/L)	26.28 ± 32.57	22.74 ± 23.01	26.32 ± 21.48	37.46 ± 64.85	0.016^∗^
AST (U/L)	37.68 ± 26.32	36.88 ± 25.98	38.45 ± 28.72	38.36 ± 20.57	0.858
ALT (U/L)	27.51 ± 20.61	28.24 ± 21.59	27.25 ± 21.46	25.81 ± 14.29	0.746
Glomerular filtration rate	109.66 ± 17.99	108.47 ± 15.22	111.03 ± 20.03	109.96 ± 20.59	0.465
Radiography					
Thrombus	149 (36.25%)	70 (35.35%)	50 (32.46%)	29 (49.15%)	0.072
Spleen diameter (mm)	201.50 ± 2.12	149.5 ± 59.12	164.75 ± 30.89	204.5 ± 68.67	0.087
Splenic vein diameter (mm)	11.40 ± 3.07	11.35 ± 3.12	10.53 ± 3.30	12.66 ± 2.64	0.453
Splenic venous flow velocity (cm/s)	15.50 ± 6.03	19.82 ± 4.64	10.03 ± 3.44	18.12 ± 3.29	< 0.001^∗∗^
Portal vein diameter (mm)	14.63 ± 4.24	15.02 ± 4.07	13.06 ± 3.24	16.96 ± 6.02	0.214
Portal vein flow velocity (cm/s)	13.72 ± 4.38	13.70 ± 4.76	13.02 ± 3.77	15.5 ± 5.43	0.653

*Note:* HCC, hepatocellular carcinoma; ALb, albumin; AST, aspartate transaminase; ALT, alanine transaminase.

Abbreviations: HBV, hepatitis B virus; INR, international normalized ratio (of prothrombin time); MELD score, model for end‐stage liver disease (natrium) score; PBC, primary biliary cholangitis; TB, total bilirubin; WBC, white blood cell (leukemia).

^∗^ *p* < 0.05; ^∗∗^ *p* < 0.01.

### 3.4. Endoscopic Features and Outcomes

Further analysis of the endoscopic examination and treatment outcomes (Table 2) revealed significant differences in the incidence of GVs (26.26% vs. 24.67% vs. 30.5%) among the three groups (*p* < 0.001). Subgroup comparisons revealed a lower rate of GVs in the S‐SPSS group than in the L‐SPSS group (*p* < 0.001) and the M‐SPSS group (*p* < 0.001). However, no significant difference in the incidence of GVs was observed between the S‐SPSS and M‐SPSS groups (*p* = 0.735). The 12‐month rebleeding rates were lower in the L‐SPSS group (3.38%) than in the S‐SPSS (14.64%) and M‐SPSS groups (16.88%) (*p* = 0.035).

**TABLE 2:** Endoscopic treatment and follow‐up of patients with SPSS.

Characteristics	With SPSS	Shunt diameter			*p*‐value
		≤ 5 mm	5–8 mm	> 8 mm	
	*n* = 411	*n* = 198	*n* = 154	*n* = 59	
EV					
Diameter (mm)	8.18 ± 3.01	8.25 ± 2.98	8.22 ± 3.19	7.82 ± 2.68	0.757
RCS	218 (53.04%)	110 (55.55%)	75 (48.70%)	33 (55.93%)	0.342
EVL (n)	6.22 ± 1.73	6.19 ± 1.53	6.45 ± 1.89	5.57 ± 1.87	0.124
EIS (ml)	16.57 ± 9.69	17.31 ± 9.10	17.27 ± 10.80	9.56 ± 4.92	0.990
With GV	108 (26.27%)	52 (26.26%)	38 (24.67%)	18 (30.50%)	< 0.001^∗∗^
GV					
Diameter (mm)	11.12 ± 4.84	10.71 ± 3.20	11.24 ± 5.09	12.09 ± 7.95	0.707
EIS (ml)	10.37 ± 8.05	8.78 ± 8.22	12.30 ± 8.12	11.20 ± 6.05	0.330
CYA (ml)	1.86 ± 1.31	1.85 ± 1.27	1.80 ± 1.398	2.20 ± 1.39	0.833
Follow‐up					
Primary eradication rate, *n* (%)	21 (5.10%)	10 (5.05%)	8 (5.19%)	3 (5.08%)	0.998
Secondary eradication rate, *n* (%)	39 (9.48%)	16 (8.08%)	19 (12.33%)	4 (6.77%)	0.299
Third eradication rates, *n* (%)	180 (43.79%)	77 (38.88%)	74 (48.05%)	29 (49.15%)	0.153
Rebleeding within 1 month, *n* (%)	20 (4.86%)	9 (4.54%)	7 (4.54%)	4 (6.77%)	0.762
Rebleeding within 6 months, *n* (%)	42 (10.21%)	17 (8.58%)	19 (12.33%)	6 (10.16%)	0.514
Rebleeding within 12 months, *n* (%)	57 (13.86%)	29 (14.64%)	26 (16.88%)	2 (3.38%)	0.035^∗^

*Note:* EIS, endoscopic sclerotherapy; CYA, cyanoacrylate; EVL, endoscopic band ligation.

Abbreviations: EV, oesophageal varices; GV, gastric varices; RCS, red colour signs.

^∗^ *p* < 0.05; ^∗∗^ *p* < 0.01.

### 3.5. Rebleeding Risk Analysis

**FIGURE 2:**
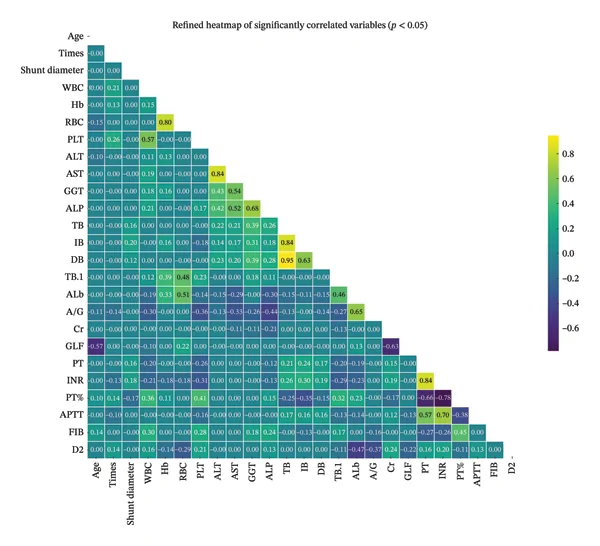
Heatmap for correlation analysis of the SPSS diameter.

Correlation analysis of the SPSS diameter (Figure 2) revealed a positive correlation with TB (*r* = 0.12, *p* = 0.023), indirect bilirubin (IB) (*r* = 0.167, *p* = 0.002), and INR (*r* = 0.130, *p* = 0.014) but a negative correlation with PT% (*r* = −0.171, *p* = 0.001). According to the results of stepwise linear regression, the linear regression equation was expressed as follows: SPSS diameter = 1.1524 + 3.3579 × INR + 0.0470 × IB, where 1.1524 is the intercept (const); 3.3579 is the coefficient for the INR, suggesting that for every unit increase in the INR, the diameter of the SPSS is expected to increase by 3.3579 mm; and 0.0470 is the coefficient for the IB, indicating an expected shunt diameter increase of 0.0470 mm for every 1 μmol/L increase in the IB.

Multivariate Cox proportional hazards regression analysis was conducted to identify independent predictors associated with the risk of rebleeding. The results are summarized in Table 3. Among the analysed variables, the shunt diameter, frequency of endoscopic surveillance, and frequency of endoscopic intervention were significantly associated with the rebleeding risk. A larger shunt diameter was significantly associated with a lower risk of rebleeding (HR = 0.230, 95% CI: 0.070–0.758; *p* = 0.016). An increased frequency of endoscopic surveillance was a strong protective factor against rebleeding (HR = 0.003, 95% CI: 0–0.148, *p* = 0.003). Similarly, more frequent endoscopic intervention was associated with a significantly reduced risk of rebleeding (HR = 0.295, 95% CI: 0.129–0.721; *p* = 0.035). Other variables, including age, sex, duration of liver cirrhosis, TB, the Child‒Pugh score, the MELD score, and the presence of HCC, were not statistically significant predictors of rebleeding (*p* > 0.05 for all).

**TABLE 3:** Multivariate Cox regression in rebleeding.

Parameters	Multivariate Cox regression			
	*p*	HR	CI lower	CI upper
Age	0.545	1.123	0.771	1.634
Sex	0.374	1.693	0.565	5.077
History of liver cirrhosis (years)	0.566	0.594	0.101	3.514
HCC	0.701	0.004	0.000	0.225
Shunt diameter	0.016	0.230	0.070	0.758
TB (μmol/L)	0.893	1.105	0.258	4.741
Endoscopic surveillance frequency	0.003	0.003	0	0.148
Endoscopic intervention frequency	0.035	0.295	0.129	0.721
Child–Pugh score	0.345	4.975	0.178	139.351
MELD score	0.619	1.318	0.444	3.909

*Note:* HCC, hepatocellular carcinoma.

Abbreviation: TB, total bilirubin.

### 3.6. Changes in Liver and Renal Function Over Time

**FIGURE 3:**
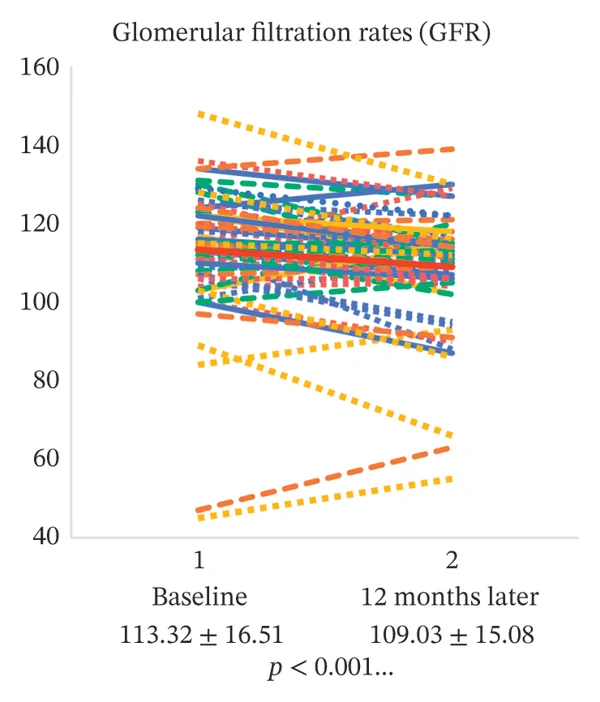
Comparison of GFRs during the 12‐month follow‐up.

A paired comparison of liver function at the 12‐month follow‐up for the 3 groups of patients revealed that TB (22.24 ± 12.78 vs. 30.17 ± 41.89; *p* = 0.007), IB (16.27 ± 9.23 vs. 20.40 ± 14.92; *p* < 0.001), and total bile acid (TBA) (24.68 ± 20.78 vs. 35.22 ± 34.77; *p* < 0.001) were significantly greater at 12 months than those at baseline (Table 4). A comparative analysis of the average glomerular filtration rate (GFR) of the 3 groups of patients revealed a significant decrease in the average GFR at 12 months compared with that at baseline (113.32 ± 16.51 vs. 109.03 ± 15.09; *p* < 0.001) (Figure 3).

**TABLE 4:** Comparison of liver function changes at baseline and after 12 months.

Variables	*X ± s*	*t*‐value	*p*‐value
TB (μmol/L)			
At baseline	22.24 ± 12.78	−2.712	0.007^∗^
12 months later	30.17 ± 41.89		
IB (μmol/L)			
At baseline	16.27 ± 9.23	−4.670	< 0.001^∗∗^
12 months later	20.40 ± 14.92		
DB (μmol/L)			
At baseline	5.88 ± 4.16	−1.570	0.118
12 months later	9.45 ± 29.75		
TP (g/L)			
At baseline	63.63 ± 8.12	−0.803	0.423
12 months later	64.15 ± 5.87		
ALb (g/L)			
At baseline	34.80 ± 4.71	−1.876	0.062
12 months later	35.46 ± 4.57		
GLb (g/L)			
At baseline	29.16 ± 6.15	1.298	0.196
12 months later	28.69 ± 5.52		
A/G			
At baseline	1.25 ± 0.32	−2.314	0.022^∗^
12 months later	1.29 ± 0.32		
ALT (U/L)			
At baseline	26.93 ± 20.75	0.743	0.459
12 months later	25.69 ± 13.14		
AST (U/L)			
At baseline	36.51 ± 26.04	−0.232	0.817
12 months later	36.98 ± 21.56		
GGT (U/L)			
At baseline	56.68 ± 70.73	0.050	0.960
12 months later	56.43 ± 68.78		
ALP (U/L)			
At baseline	115.25 ± 65.26	−0.012	0.990
12 months later	115.29 ± 66.23		
TBA (μmol/L)			
At baseline	24.68 ± 20.78	−3.719	< 0.001^∗∗^
12 months later	35.22 ± 34.77		

*Note:* ALb, albumin; GLb, globulin; AST, aspartate transaminase; A/G, albumin/globulin ratio; ALT, alanine transaminase; ALP, alkaline phosphatase.

Abbreviations: DB, direct bilirubin; GGT, gamma‐glutamyl transferase; IB, indirect bilirubin; TP, total protein; TB, total bilirubin; TBA, total bile acid.

^∗^ *p* < 0.05.

^∗∗^ *p* < 0.01.

## 4. Discussion

### 4.1. Detection of SPSSs in Patients With EGVB

The present study revealed that the incidence of SPSSs in patients with cirrhosis primarily seeking treatment for EGVB was 94.26%, indicating that almost all patients with oesophagogastric varices also had an SPSS. This percentage is much higher than the previously reported SPSS prevalence of approximately 60% [6] and even higher than the 40% reported when ultrasound is used as a diagnostic basis [7, 8]. This discrepancy may be related to the use of portal venous imaging techniques for diagnosis [9]. Silvia et al. suggested that CTA is the gold standard for detecting SPSSs because it can track the origin, process, and exit of the shunt through 3D reconstruction [10, 11]. Simón‐Talero et al. and Bandali et al. reported [6, 12] that the most common types of SPSSs are SRSs (42%) and UVSs (54%). Nevertheless, the present data indicated that SRSs accounted for only 1.14% and UVSs accounted for 3.89% in cirrhotic patients primarily seeking treatment for EGVB. Moubarak reported that GVs are more common in patients with GRSs [13], which is consistent with the present finding that GRSs accounted for 78.89%.

Furthermore, Arora et al. reported that GRSs mainly involve the left retroperitoneal vein and are related to dilation of the left renal vein [14]. These SPSSs may originate from pre‐existing small portal systems or the adrenal and perirenal venous systems. In patients with GRSs, portal blood mainly flows through the short gastric veins and posterior gastric veins into the gastric fundus, where it forms an isolated GV, which then circulates through the GRS to the left renal vein. However, the present findings revealed only 2 patients in which the GRS formed an isolated GV. Moreover, in 99.41% of patients, varices appeared in both the oesophagus and stomach simultaneously, suggesting that patients with EGVB have multiple portal collateral pathways and high portal pressure.

### 4.2. Relationship Between SPSSs and Liver Function in Patients With EGVB

The present study also revealed that liver function in cirrhotic patients was negatively correlated with the SPSS diameter and significantly correlated with the intrahepatic biliary duct and SPSS diameter. An increase in the diameter of the SPSS was accompanied by worsening liver function. The baseline liver function was related to the SPSS diameter, with the MELD score and TB increasing in the L‐SPSS group, suggesting that a larger shunt diameter is associated with poorer liver function. However, there was no statistically significant difference in the Child‒Pugh score (*p* = 0.67), which is consistent with the findings reported by Greinert [15], showing an independent correlation between the presence of an SPSS and the MELD score. In a study by Nardelli on the relationship of SPSSs and sarcopenia with HE prognosis and the nontransplant survival rate, the MELD score and sarcopenia score were reported to be the only variables significantly associated with the nontransplant survival rate [16].

Additionally, in Greinert’s study [15], the Child‒Pugh score showed no statistical difference. In Tarantino’s study [16] on the relationship among SRSs, complications, and cirrhosis prognosis, the Child‒Pugh score was not statistically significant in evaluating the prognosis of patients with SRSs. Analysis of baseline data revealed that the presence of shunts adversely affects liver function. A comparison of liver function at the 12‐month follow‐up revealed that the TB, IB, direct bilirubin (DB), and TBA levels tended to increase compared with the baseline levels, whereas the alanine transaminase (ALT), aspartate transaminase (AST), γ‐glutamyl transferase (GGT), and alkaline phosphatase (ALP) levels did not significantly differ from the baseline levels. Moreover, there was a significant difference in the comparison of the TB, IB, and TBA levels before and after follow‐up. As the SPSS acts as a compensatory mechanism for portal hypertension, diverted blood flow reduces liver perfusion, preventing more blood from passing through the liver, which suggests that the liver function damage is due primarily to elevated IB levels [17]. Kothari et al. reported more severe liver function impairment in patients with L‐SPSS in a prospective study [18]. After conducting a multicentre survey, Simón‐Talero et al. concluded that the presence and size of the SPSS increase with liver function impairment and that the prevalence of SPSSs increases with worsening liver function [6]. In the present study, the presence of an SPSS was more likely to lead to worsening liver function. Furthermore, Tatsumi et al. demonstrated that liver function significantly improves and that the liver stiffness decreases after intervention and embolization of an SPSS [19].

### 4.3. Relationship Between SPSSs and HE in Patients With EGVB

Previous studies have demonstrated that portosystemic shunting plays a significant role in the development of HE. Several reports [15, 20] have suggested that the presence of an SPSS may increase the risk of HE. However, in the present study, only six patients developed overt hepatic encephalopathy (OHE)—two in the S‐SPSS group, three in the M‐SPSS group, and one in the L‐SPSS group. Notably, one patient developed a spontaneous intrahepatic shunt, which, despite its small diameter, was associated with recurrent episodes of HE. Previous case reports [21] have also indicated that such spontaneous intrahepatic shunts may predispose patients to HE.

Importantly, the present study focused primarily on patients with GRSs, which limits the generalizability of the findings regarding the associations between various SPSS subtypes and HE. According to the literature [11], different SPSS types may confer distinct risks for HE and other complications. For example, SRSs and UVSs are more commonly associated with HE, whereas GRSs are more frequently linked to variceal bleeding, with a lower incidence of both HE and PVT. In the present study, GRSs accounted for the majority of cases (78.89%), and the overall incidence of HE was low, which is consistent with prior reports. Nevertheless, owing to the limited sample size, the present findings warrant further validation in larger, multicentre studies to better elucidate the specific impact of the SPSS subtype on the risk of HE.

### 4.4. Relationship Between SPSSs and Rebleeding in Patients With EGVB

The present study demonstrated that patients with L‐SPSS had a lower risk of rebleeding within 12 months, despite being more likely to present with gastric varices (GV). Notably, a larger shunt diameter was independently associated with a reduced rebleeding risk, with each millimeter increase corresponding to a 77% decrease in the hazard. The predominant shunt types observed were GRSs and SRSs, both of which are known for their substantial haemodynamic impact. Other SPSS variants, such as UVSs, were underrepresented. Given that the anatomical configuration influences shunt flow and portal pressure differently, the observed protective effect may reflect the dominant subtypes in this cohort rather than being generalizable to all SPSS types.

We also found that both the frequency of endoscopic surveillance and the number of endoscopic interventions were significantly associated with lower rebleeding rates. Patients who underwent more frequent endoscopic monitoring had an almost complete reduction in the rebleeding risk, and those receiving timely interventions also substantially benefited. These findings emphasize the importance of regular surveillance and proactive treatment in managing patients with portal hypertension, particularly those with known SPSSs.

Nevertheless, the mechanisms by which different SPSS configurations influence variceal bleeding remain incompletely understood. Further studies with larger cohorts and balanced subtype representations are needed to delineate the haemodynamic profiles and clinical implications of various SPSS patterns.

### 4.5. Relationship Between SPSSs and Renal Function in Patients With EGVB

The present study revealed that the diameter and type of SPSS are not constant and can change with disease progression. Over the 12‐month follow‐up, a significant decrease in the GFR was observed, which is consistent with findings by ZHANG, who linked SPSSs to HRS and reported that an SPSS diameter > 1.5 cm is an independent risk factor for HRS [22]. The SPSS may provide a direct path for vasodilator substances into the circulation; a larger SPSS indicates that more vasodilator substances enter the circulation with less inactivation by the liver, exacerbating the renal response. Reduced portal blood flow from the SPSS causes renal sympathetic activation, leading to renal artery constriction and consequent renal perfusion reduction, ultimately causing renal dysfunction and triggering HRS formation.

The present study had several limitations, as it was a single‐centre retrospective study with a short duration. Future multicentre randomized controlled studies are needed to confirm the clinical significance of SPSSs [23].

## 5. Conclusions

In conclusion, the detection rate for SPSSs in patients with EGVB is high. The present study suggested that the SPSS has a dual role: it protects against EGVB and impacts liver and kidney functions to various degrees. The different types of SPSSs are critical factors in the various complications observed during decompensated cirrhosis. Cirrhotic patients with EGVB are recommended to undergo portal vein reconstruction to evaluate the SPSS diameter, direction, and involvement in the formation of oesophageal and GVs.

## Author Contributions

Derun Kong contributed to revising, editing, final approval, and funding acquisition; Zhaoyi Chen contributed to drafting the article; Jing Li and Xi Wang contributed to data curation and formal analysis; Yaxian Kuai and Huixian Li contributed to investigation; Xuecan Mei contributed to project administration.

## Funding

This work was supported by the National Natural Science Foundation of China (No. 82270623), the Sixth Batch of Health Appropriate Technology Promotion Project in Anhui Province (No. SYJS202103), and the Anhui Province Key Research and Development Program Project (No. 2022e07020048).

## Ethics Statement

The study was approved by the Committee on Medical Ethics of The First Affiliated Hospital of Anhui Medical University (Quick PJ 2024‐02‐45) and conducted in accordance with the Declaration of Helsinki. Written informed consent was obtained from the participants.

## Conflicts of Interest

The authors declare no conflicts of interest.

## Supporting Information

STROBE checklist.

## Supporting information


**Supporting Information** Additional supporting information can be found online in the Supporting Information section.

## Data Availability

The datasets used and/or analysed during the current study are available from the corresponding author on reasonable request.
